# Fe-Based Amorphous Microflakes with Deformation-Induced Nanoscale Heterogeneity Prepared by Pulse-Discharge Atomization and Ball Milling for Broadband Microwave Absorption

**DOI:** 10.3390/nano16191242

**Published:** 2026-10-01

**Authors:** Chekai Liu, Xinghua Wang, Zonghai Zhao, Rui Ding, Yanming Wu, Chenghui Hu, Wei Xue, Jingwu Liu, Xinghao Wei

**Affiliations:** 1 Luoyang Ship Material Research Institute, Luoyang 471023, China; 2 China Ship Research and Development Academy, Beijing 100101, China

**Keywords:** pulse discharge atomization, ball milling, Fe-based amorphous alloy, microflakes, nanoscale heterogeneity, microwave absorption

## Abstract

Fe-based amorphous alloys are attractive for microwave attenuation because they combine soft-magnetic behavior, relatively high electrical resistivity compared with compositionally similar crystalline alloys, and flexible structural design. A persistent challenge is to achieve strong attenuation and broad bandwidth at a small matching thickness without relying on complex multiphase architectures. Fe_85_P_9_C_2_B_2_Si_2_ amorphous powders were prepared by pulse-discharge atomization and the fine spherical fraction was subsequently flattened by controlled wet-ball milling. The atomized powders remain predominantly amorphous, whereas milling produces high-aspect-ratio microflakes together with localized approximately 5 nm band-like heterogeneous regions containing limited nanocrystalline order. Relative to the spherical powders, FP5 shows stronger dielectric and magnetic responses. At 16.9 GHz and a thickness of 1.7 mm, the attenuation constant reaches 235.9 m^−1^ and the normalized input-impedance magnitude is 0.999, indicating that strong intrinsic attenuation coincides with nearly ideal impedance matching. FP5 consequently exhibits a minimum reflection loss of −53.5 dB and an effective absorption bandwidth of 6.2 GHz (11.8–18.0 GHz) at 1.85 mm. The results indicate that the high-aspect-ratio morphology is the better-supported primary structural contribution to the improved absorption, while local amorphous/nanocrystalline heterogeneity may provide an additional contribution.

## 1. Introduction

The rapid expansion of wireless communication, radar, sensing, and portable electronic systems operating in the gigahertz (GHz) range has intensified electromagnetic interference and microwave pollution. Accordingly, advanced microwave absorbers are expected to combine strong attenuation, a broad effective absorption bandwidth (EAB), a small matching thickness, and stable electromagnetic properties [1,2]. For shipborne and marine platforms, practical microwave absorbers must further reconcile broadband attenuation with thin profiles, weight constraints, engineering processability, and environmental adaptability [3]. Magnetic metallic and alloy powders are attractive because they can provide both dielectric and magnetic loss channels; however, conventional crystalline metallic powders often suffer from eddy-current loss and skin-depth limitation at high frequencies, whereas ferrites generally exhibit lower saturation magnetization [4,5].

Fe-based amorphous alloys are typically Fe-rich compositions containing glass-forming metalloids such as B, P, C, and Si, with other elements sometimes added to adjust glass-forming ability and magnetic response [6,7]. Formation of the amorphous state depends on both alloy chemistry and cooling history: the composition must resist crystallization, and solidification must be sufficiently rapid to suppress long-range atomic ordering. In Fe_85_P_9_C_2_B_2_Si_2_, the combined additions of P, B, C, and Si promote glass formation, while rapid quenching during pulse-discharge atomization helps preserve the disordered structure. Fe-based metallic glasses also offer high strength and useful wear/corrosion resistance [8,9]. For microwave absorption, however, bulk composition is only one design variable; particle size, morphology, magnetic anisotropy, and local structural heterogeneity also influence electromagnetic penetration, polarization, impedance matching, and energy dissipation [10,11,12,13].

Several routes are available for preparing amorphous alloy powders. Gas and water atomization provide high throughput, whereas mechanical alloying can directly generate amorphized powders from blended precursors [14]. Thermal treatment offers another means of tuning electromagnetic behavior, although partial crystallization must be carefully controlled [15]. Wet-chemical routes have recently produced nanoscale amorphous Co–Fe absorbers with good compositional control [16]. These approaches are effective, but they differ in precursor chemistry, equipment requirements, and the degree to which particle size and post-processing effects can be separated. A route that converts a pre-alloyed bulk composition directly into amorphous powders spanning a broad size range is therefore attractive for studying size- and morphology-dependent absorption within a single alloy system.

Pulse-discharge atomization provides such a route by converting a conductive bulk alloy directly into fine powders through repeated electrical discharges in a dielectric liquid. The plate–rod electrode configuration, discharge behavior, and formation mechanism of Fe-based amorphous powders have been described in our previous study [17]. Transient plasma heating causes local melting and ejection, followed by rapid cooling in the dielectric medium [18,19,20,21,22]. An important feature of this process is that a single atomized batch contains a wide range of particle sizes while retaining the nominal alloy composition, enabling different size fractions to be selected before any subsequent morphology modification. This provides a convenient basis for separating the effect of particle-size refinement from the effect of post-milling flake formation.

Flake-like morphology is also widely used to improve the high-frequency response of magnetic powders. Increasing the aspect ratio changes the demagnetizing field and magnetic anisotropy, increases the effective interaction area, and can improve the balance between dielectric and magnetic response [23]. Recent FeSiCr studies further show that flake morphology and heterogeneous interfaces can broaden the absorption band [24,25]. In amorphous alloys, ball milling may additionally generate highly localized deformation structures. Because such local features occupy only a small fraction of the material, their contribution must be distinguished from the much larger, population-wide change in particle morphology.

In this work, Fe_85_P_9_C_2_B_2_Si_2_ amorphous powders were first prepared by pulse-discharge atomization. Two spherical fractions, SP15 and SP5, were selected from the same atomized batch, and SP5 was then converted into the flake sample FP5 by controlled wet-ball milling. This sequence allows the SP15 → SP5 comparison to emphasize particle-size refinement and the SP5 → FP5 comparison to capture the additional effect of flake formation within the same alloy chemistry. Phase structure, thermal stability, morphology, local nanostructure, magnetic properties, electromagnetic parameters, and microwave absorption were examined together with impedance matching, attenuation, dielectric-relaxation, and magnetic-loss analyses.

## 2. Materials and Methods

Alloy ingots with a nominal composition of Fe_85_P_9_C_2_B_2_S_i2_ (at.%) were prepared by induction melting mixtures of Fe (99.9 mass%), C (99.9 mass%), Fe-P pre-alloy (22.1 mass% P), and Fe-B pre-alloy (20 mass% B) under an argon atmosphere. Plates (40 mm × 10 mm × 2 mm) and rods (3 mm in diameter) were fabricated from the ingots by copper-mold spray casting and used as the two electrodes for pulse-discharge atomization. The spark-discharge apparatus, electrode arrangement, kerosene dielectric, discharge-parameter definitions, and general powder-preparation procedure followed our previously reported method [17]. The as-produced powders were ultrasonically cleaned with dichloromethane at least three times to remove residual dielectric fluid.

The as-atomized powders were sieved using nylon sieves to obtain a coarse spherical fraction with an average particle size of approximately 15 μm (SP15) and a fine spherical fraction with an average particle size of approximately 5 μm (SP5). SP5 was subsequently wet-ball-milled in an ND8-04L planetary ball mill (Nanjing Nanda Tianzun Electronics Co., Ltd., Nanjing, China) using an Al_2_O_3_ ceramic vial and 5 mm Al_2_O_3_ ceramic balls. Anhydrous ethanol was used as the milling medium. The rotation speed was 400 rpm and the powder-to-ball mass ratio was 1:10. Milling was conducted in four 30 min cycles (2 h total effective milling time), with cooling intervals between cycles. After milling, the slurry was collected and the powder was dried before further characterization.

Phase structure was characterized by X-ray diffraction (XRD; Rigaku D/max-2200 PC) using Cu Kα radiation. Particle-size distributions were measured using a laser particle-size analyzer (Mastersizer 2000). Morphology and nanoscale structure were examined by scanning electron microscopy (SEM; JEOL JSM-7500) and transmission electron microscopy (TEM; JEM-2100F), including high-resolution TEM (HRTEM) and selected-area electron diffraction (SAED). Thermal behavior was evaluated by differential scanning calorimetry (DSC; PerkinElmer DSC 8000) at a heating rate of 20 K/min. Room-temperature magnetic properties were measured using a vibrating sample magnetometer (VSM; Lake Shore 7404).

For electromagnetic measurements, the powders were mixed with paraffin wax at a 1:1 mass ratio and pressed into coaxial toroidal specimens with an outer diameter of 7 mm and an inner diameter of 3 mm. The complex permittivity and permeability were measured from 2 to 18 GHz using an Agilent N5234A vector network analyzer. One toroidal specimen was measured for each powder condition. Reflection loss was calculated from the measured electromagnetic parameters using transmission-line theory.

## 3. Results

### 3.1. Phase Structure, Thermal Stability and Static Magnetic Properties

**Figure 1:**
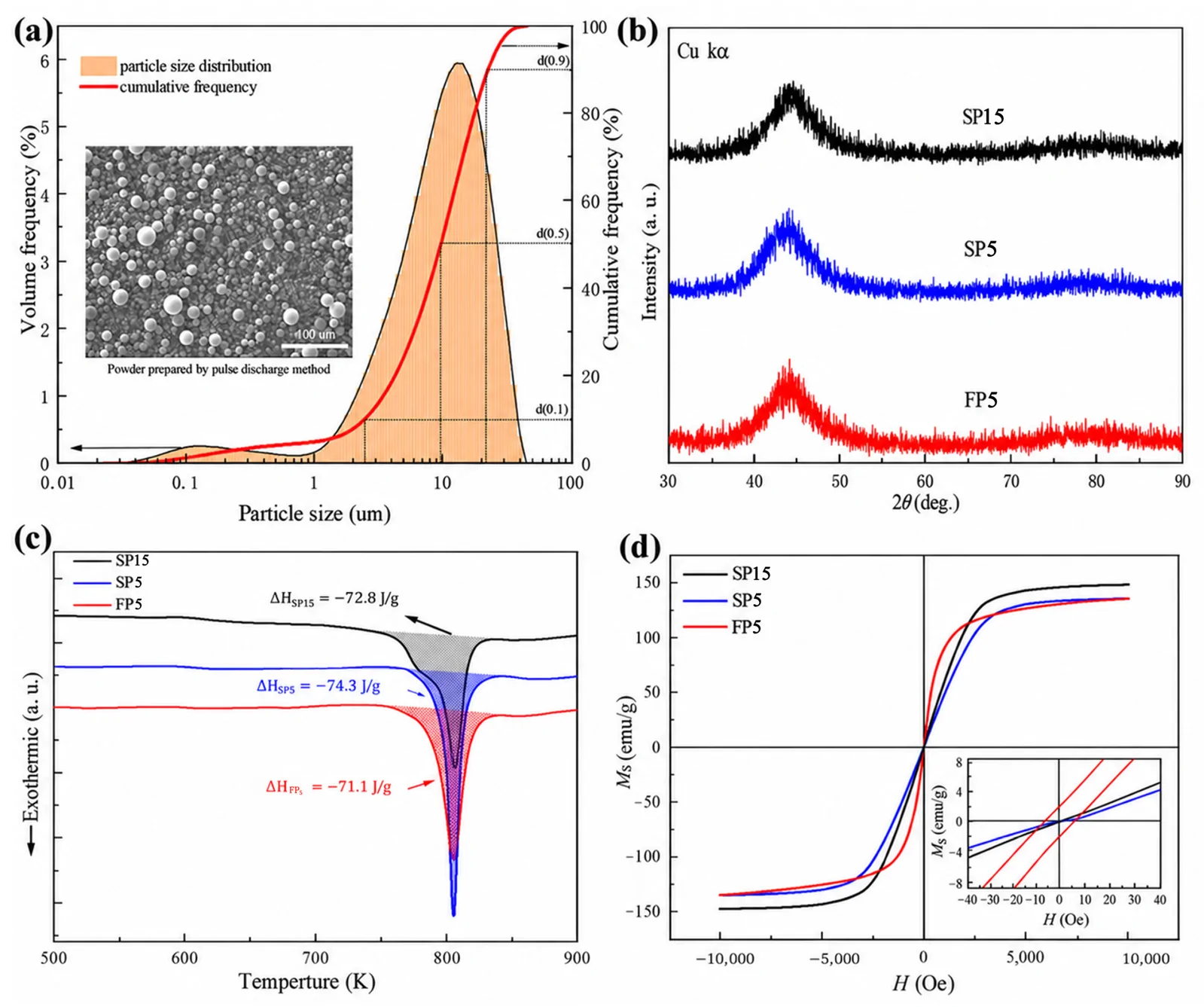
(**a**) Particle-size distribution and SEM image of the as-atomized powder, (**b**) XRD patterns, (**c**) DSC curves at a heating rate of 20 K/min and (**d**) room-temperature hysteresis loops of SP15, SP5 and FP5.

Pulse-discharge atomization produced nearly spherical, mutually separated Fe-based powders without obvious agglomeration or interparticle bonding (Figure 1a). The particle-size distribution extends from approximately 25 nm to 40 μm and is bimodal, with peaks near 150 nm and 13 μm. The characteristic sizes d(0.1), d(0.5), and d(0.9) are 2.4, 9.5, and 21.4 μm, respectively, corresponding to a distribution span of 2.00. The broad, bimodal distribution is consistent with the coexistence of fine fragments and micron-scale molten droplets during pulse-discharge atomization.

The XRD patterns of SP15, SP5, and FP5 are shown in Figure 1b. All three samples exhibit a broad diffuse halo centered at approximately 2θ = 44°, with no detectable crystalline diffraction peaks, indicating predominantly amorphous structures at the XRD detection scale. The DSC curves (Figure 1c) show crystallization enthalpies of 72.8, 74.3, and 71.1 J/g for SP15, SP5, and FP5, respectively. The small decrease from SP5 to FP5 indicates that most of the amorphous matrix is retained after milling, while allowing for limited structural relaxation and localized crystallization [26].

The room-temperature hysteresis loops are presented in Figure 1d. SP15 and SP5 show typical soft-magnetic behavior, with coercivities of 1.15 and 1.58 Oe, respectively. The saturation magnetization decreases from 145 emu/g for SP15 to approximately 134 emu/g for SP5 and FP5, while the coercivity of FP5 increases to 6.24 Oe. The increase after milling is consistent with the combined influence of deformation strain, shape anisotropy, and additional local pinning sites [27,28]. FP5 nevertheless retains a substantial magnetic response over the GHz range.

### 3.2. Morphological Evolution and Local Nanoscale Heterogeneity

**Figure 2:**
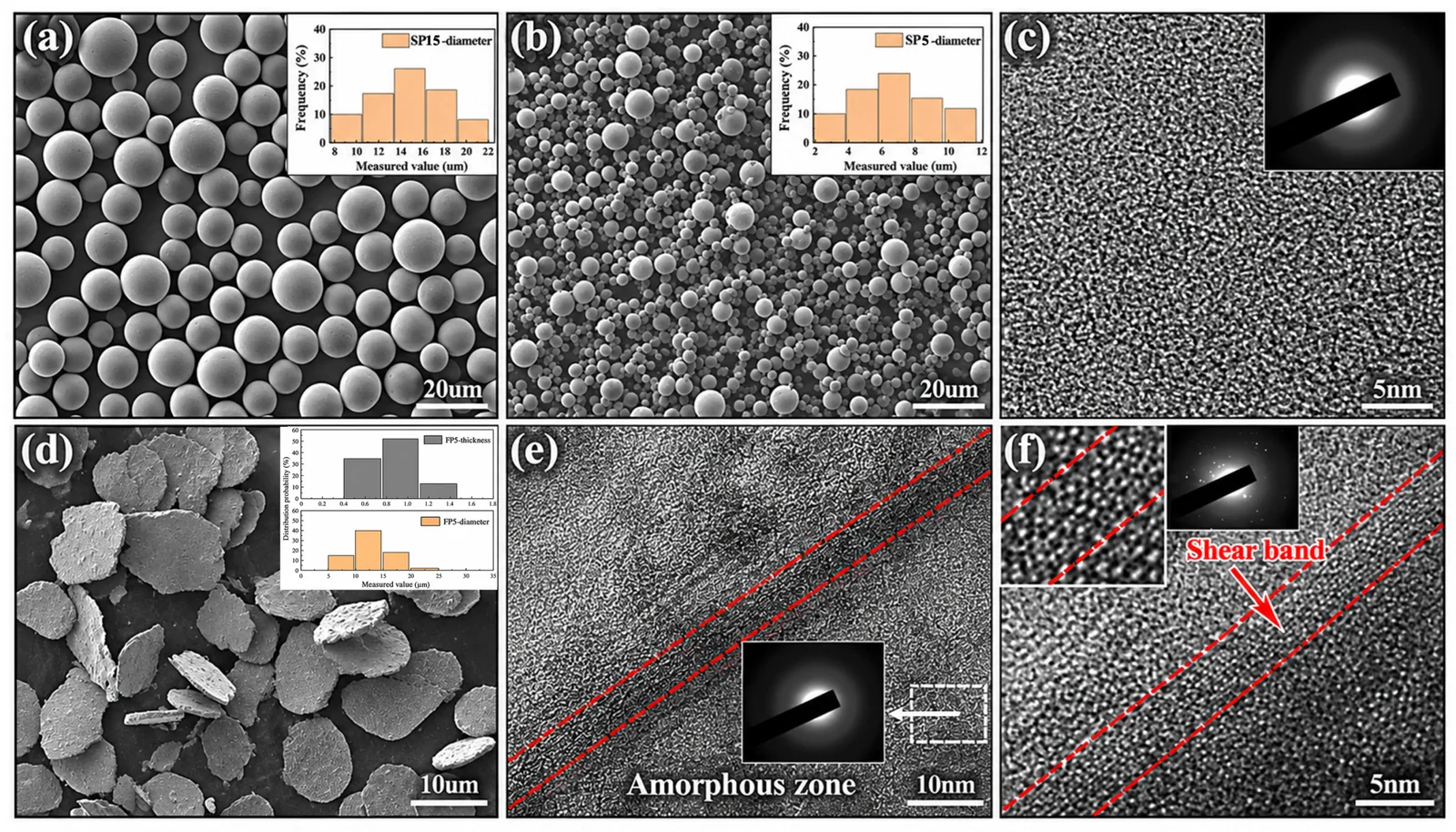
SEM images and corresponding size statistics of (**a**) SP15, (**b**) SP5 and (**d**) FP5, and HRTEM images and SAED patterns of (**c**) SP5 and (**e**,**f**) FP5.

The surface morphologies and nanoscale structures of SP15, SP5, and FP5 are shown in Figure 2. SP15 and SP5 display spherical or near-spherical morphologies with relatively smooth surfaces (Figure 2a,b). Particle-size statistics obtained from the SEM images give ranges of 11–19 μm for SP15 and 2–7 μm for SP5, consistent with the nominal sample designations. The HRTEM image and SAED pattern of SP5 show no resolvable lattice fringes or discrete diffraction spots, further supporting the predominantly amorphous state of the fine spherical powder (Figure 2c).

After ball milling, SP5 is transformed into flake-shaped FP5 particles with thicknesses of approximately 0.4–1.4 μm and lateral dimensions of 8–18 μm (Figure 2d), corresponding to an approximate aspect-ratio range of 6–45. The flakes remain largely separated and retain rounded edges, indicating that the selected milling condition favors plastic flattening rather than extensive fragmentation or cold welding. Their dimensions are also consistent with deformation of the original fine spherical particles rather than aggregation of multiple particles.

HRTEM images of FP5 show an amorphous matrix containing narrow band-like regions with local lattice fringes (Figure 2e,f). These regions are approximately 5 nm wide and are accompanied by spot-like features in the SAED pattern, indicating limited nanocrystalline order within selected deformed regions. Their morphology is consistent with shear-band-associated structural heterogeneity reported in metallic glasses [28]. Because the observation is local, it is used here as evidence that milling modifies the nanoscale structure of selected regions rather than as a measure of the bulk nanocrystalline fraction.

### 3.3. Electromagnetic Parameters and Loss Analysis

**Figure 3:**
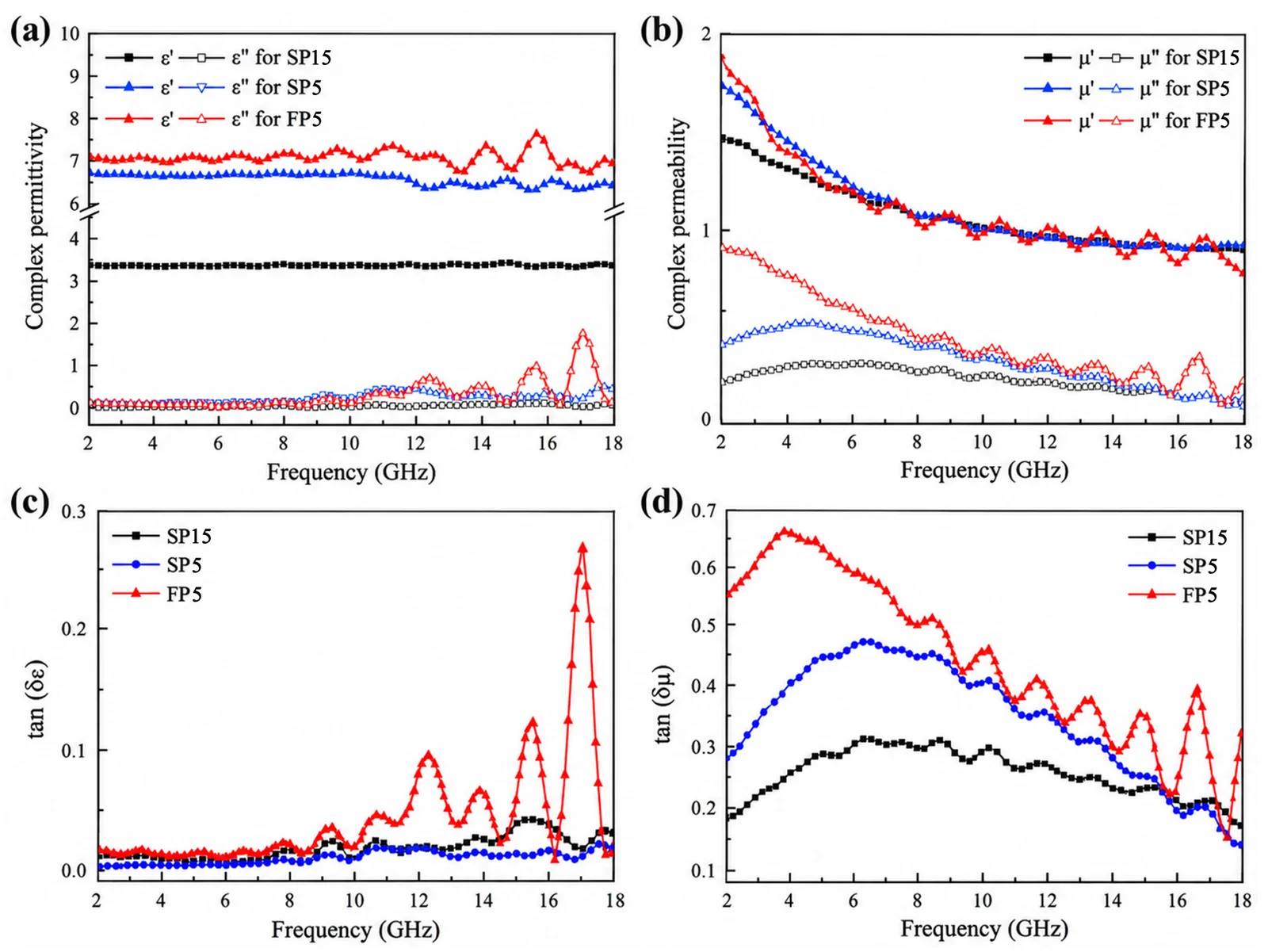
Frequency-dependent electromagnetic properties of SP15, SP5 and FP5: (**a**) complex permittivity, (**b**) complex permeability, (**c**) dielectric loss tangent and (**d**) magnetic loss tangent.

Figure 3 summarizes the frequency-dependent complex permittivity, complex permeability, and corresponding loss tangents. FP5 shows higher ε′ and ε″ than the spherical powders over most of the measured band, and tanδε rises markedly in the high-frequency region (Figure 3a,c). The μ″ and tanδμ values are likewise enhanced over a substantial frequency interval (Figure 3b,d). Thus, converting SP5 into the flake morphology strengthens both dielectric and magnetic dissipation, particularly above approximately 10 GHz.

Particle-size refinement also favors microwave penetration by reducing the characteristic dimension over which eddy-current shielding develops [29]. This provides a plausible explanation for part of the improvement from SP15 to SP5. The additional change from SP5 to FP5 coincides with the population-wide transition from spherical particles to high-aspect-ratio flakes and therefore requires consideration of polarization, magnetic anisotropy, impedance matching, and attenuation together.

The magnetic response also changes with particle size and morphology. The flake geometry alters the demagnetizing field and magnetic anisotropy [23,27], while milling-induced strain and local structural heterogeneity may affect domain-wall pinning and resonance behavior [28]. The measured μ″ and tanδμ spectra therefore suggest contributions from more than one magnetic-loss process.

#### Quantitative Electromagnetic-Loss Analysis

Microwave absorption requires both efficient entry of the incident wave into the absorber and sufficient dissipation after entry. Accordingly, the attenuation constant α and normalized input impedance Z_in_/Z_0_ were calculated from the measured complex permittivity and permeability. The attenuation constant was evaluated using

$$\alpha =\frac{\sqrt{2}\pi f}{c}\sqrt{\left(\mu^{\prime \prime }\varepsilon^{\prime \prime }-\mu^{\prime }\varepsilon^{\prime }\right)+\sqrt{{\left(\mu^{\prime \prime }\varepsilon^{\prime \prime }-\mu^{\prime }\varepsilon^{\prime }\right)}^{2}+{\left(\mu^{\prime }\varepsilon^{\prime \prime }+\mu^{\prime \prime }\varepsilon^{\prime }\right)}^{2}}}$$

**Figure 4:**
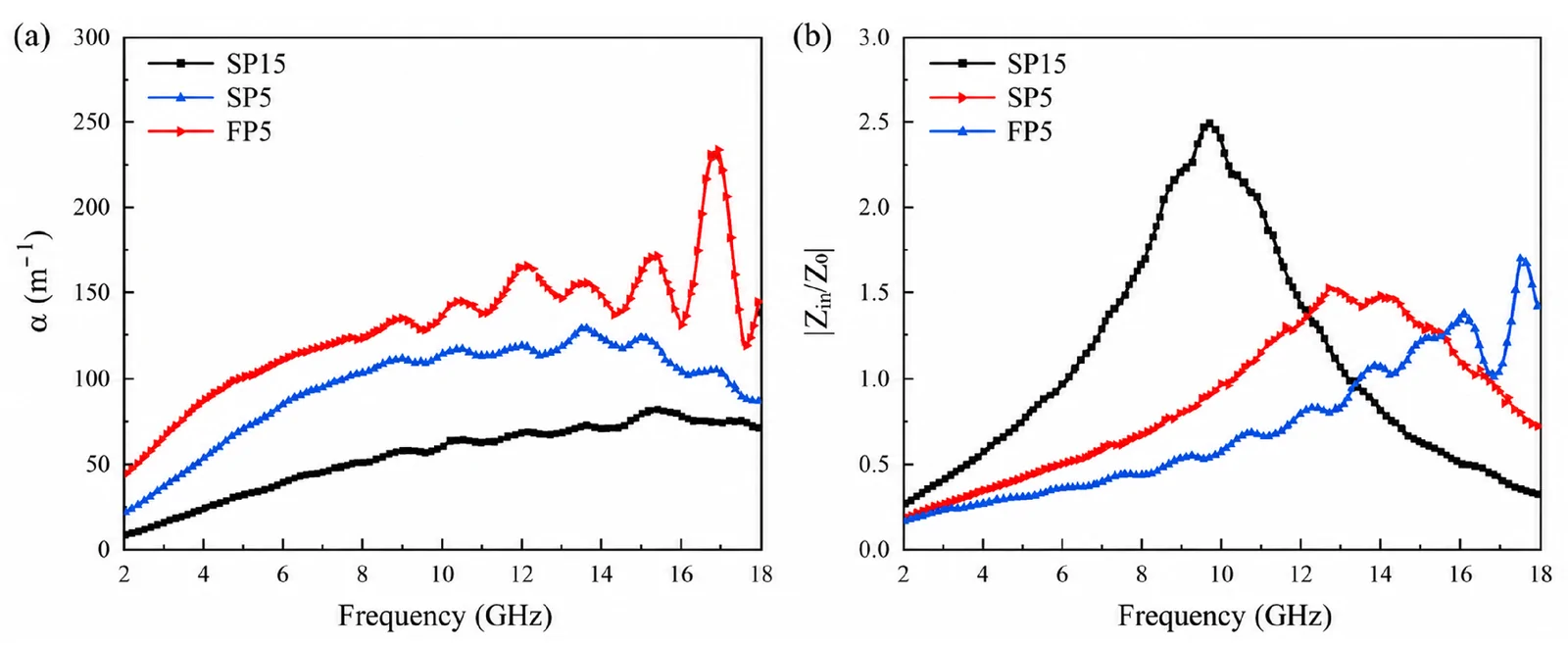
Quantitative analysis of microwave attenuation and impedance matching: (**a**) frequency dependence of the attenuation constant α for SP15, SP5 and FP5; (**b**) normalized input-impedance magnitude |Z_in_/Z_0_| calculated at representative thicknesses of 4.0 mm (SP15), 2.2 mm (SP5) and 1.7 mm (FP5).

As shown in Figure 4a, the calculated α increases progressively from SP15 to SP5 and FP5. At the frequencies corresponding to the strongest absorption of each sample, α is approximately 58.1 m^−1^ for SP15 (9.2 GHz), 129.8 m^−1^ for SP5 (13.5 GHz), and 235.9 m^−1^ for FP5 (16.9 GHz). The much larger value for FP5 confirms that flake formation substantially enhances intrinsic electromagnetic dissipation in the high-frequency region.

The normalized input impedance was calculated from

$$\frac{Z_{\mathrm{in}}}{Z_{0}}=\sqrt{\frac{\mu_{r}}{\varepsilon_{r}}}\tanh\left[j\frac{2\pi \mathrm{fd}}{c}\sqrt{\mu_{r}\varepsilon_{r}}\right]$$

Figure 4b compares the normalized input-impedance magnitude at representative matching thicknesses. At the corresponding absorption minima, Z_in_/Z_0_ changes from approximately 2.126 + j0.681 for SP15 to 1.409 − j0.175 for SP5 and 0.999 + j0.004 for FP5. The associated magnitudes are 2.232, 1.420, and 0.999, respectively. The FP5 value is essentially unity and its imaginary part is close to zero at 16.9 GHz, indicating nearly ideal impedance matching at the frequency where the attenuation constant is also highest.

The Cole–Cole behavior provides complementary information on dielectric relaxation. As shown in Supplementary Figure S1a, FP5 exhibits several distorted high-frequency arcs rather than a single ideal semicircle, consistent with multiple or distributed relaxation processes after flattening. These processes may be associated with the increased interfacial area, deformation-related structural fluctuations, surface defects, or other local polarization sites. Because the effective electrical conductivity of the powder/paraffin specimens was not measured independently, the present data are not used to quantitatively separate polarization loss from conduction loss.

The role of eddy-current loss was evaluated using

$$C_{0}=\frac{\mu^{\prime \prime }}{{\mu^{\prime }}^{2}f}$$

The magnetic-loss mechanism was further assessed using the eddy-current coefficient C_0_. A nearly frequency-independent C_0_ would be expected if eddy-current loss dominated over a broad band. Supplementary Figure S1b shows instead that C_0_ varies strongly with frequency for all three samples and exhibits additional fluctuations for FP5. Thus, eddy-current loss alone cannot account for the magnetic attenuation, and resonance-related processes are also likely to contribute.

### 3.4. Microwave Absorption Performance

The reflection loss (RL) values were calculated from the measured electromagnetic parameters using transmission-line theory. Defining the normalized input impedance as Z_N_ = Z_in_/Z_0_,

$$Z_{N}=\sqrt{\frac{\mu_{r}}{\varepsilon_{r}}}\tanh\left[j\frac{2\pi \mathrm{fd}}{c}\sqrt{\mu_{r}\varepsilon_{r}}\right]$$

$$\mathrm{RL}=20\log_{10}\left|\frac{Z_{N}-1}{Z_{N}+1}\right|$$

where Z_in_/Z_0_ is the normalized input impedance, ε_r_ and μ_r_ are the relative complex permittivity and permeability, *f* is the frequency, d is the absorber thickness, and c is the speed of light in vacuum. RL < −10 dB corresponds to more than 90% absorption of incident power, and the corresponding frequency interval is defined as the effective absorption bandwidth (EAB).

**Figure 5:**
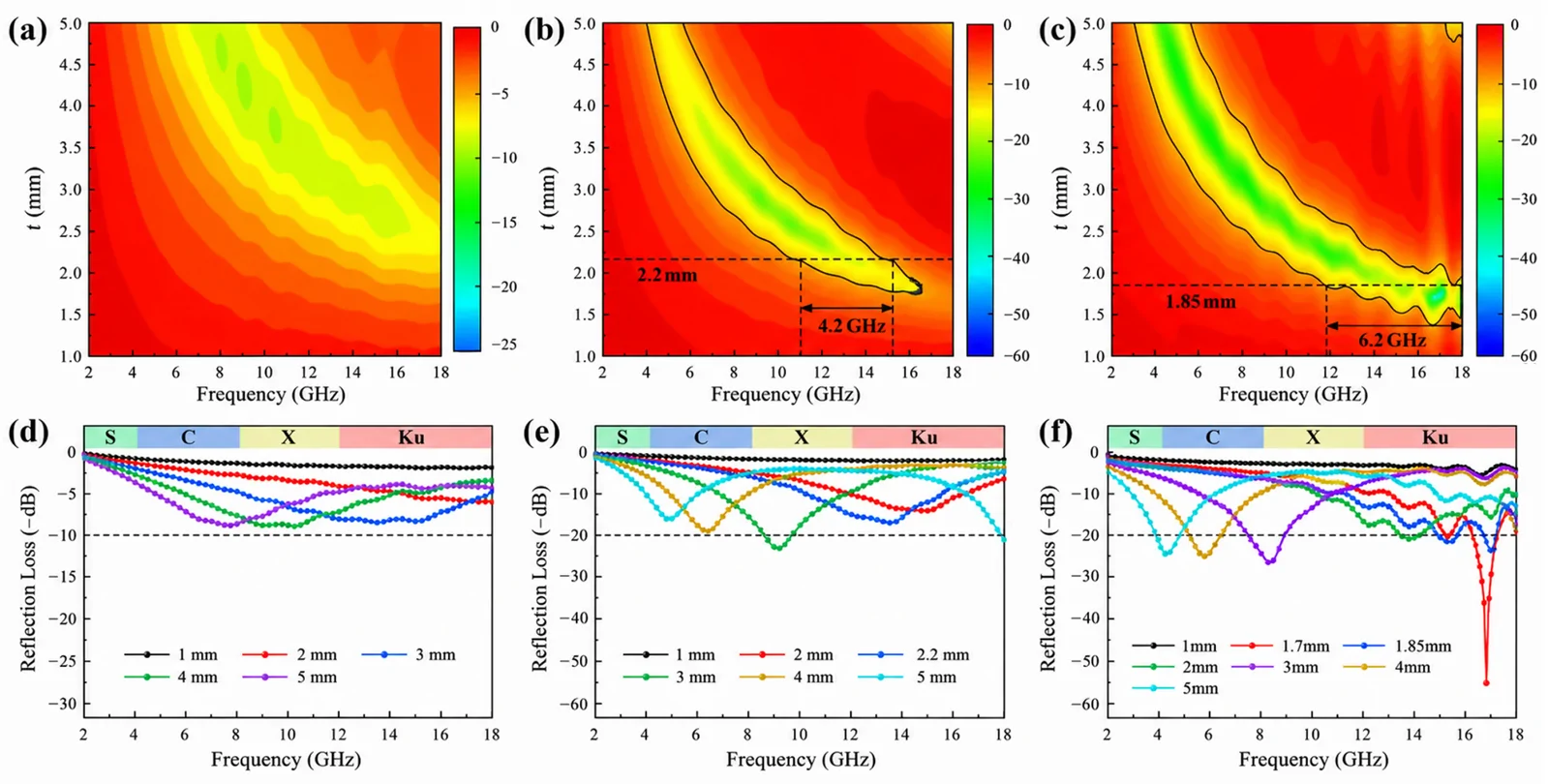
Two-dimensional projection maps and frequency-dependent reflection loss curves of (**a**,**d**) SP15, (**b**,**e**) SP5 and (**c**,**f**) FP5 at different absorber thicknesses.

As shown in Figure 5a,d, SP15 exhibits weak microwave absorption and does not reach −10 dB under the investigated conditions. Reducing the particle size to SP5 markedly improves the response: the minimum RL reaches −14.7 dB at 13.5 GHz with a matching thickness of 2.2 mm, and the EAB reaches 4.23 GHz. The improvement is consistent with better microwave penetration and the associated changes in electromagnetic parameters after particle-size refinement.

FP5 exhibits the strongest microwave absorption among the three samples. The minimum RL reaches −53.5 dB at 16.9 GHz with a matching thickness of 1.7 mm, and an EAB of 6.2 GHz is obtained at 1.85 mm, covering 11.8–18.0 GHz (Figure 5c,f). At the RL minimum, FP5 simultaneously exhibits the largest attenuation constant (235.9 m^−1^) and almost ideal impedance matching (|Z_in_/Z_0_| = 0.999; Figure 4). This coincidence explains the sharp improvement relative to SP5 more directly than considering either dielectric or magnetic loss alone.

Representative Fe-based absorbers from the literature are compared in Table 1; differences in filler fraction, matrix, sample geometry, and matching condition should be considered when comparing absolute RL and bandwidth values.

## 4. Discussion

The sequential sample design helps separate the main structural factors controlling the microwave response. SP15 and SP5 have the same nominal composition and similar spherical morphology, so their different absorption behavior mainly reflects particle-size refinement. The smaller SP5 particles show improved high-frequency penetration and a stronger magnetic-loss response, which is consistent with the reduction in RL from SP15 to SP5. The much larger change occurs after SP5 is flattened into FP5. Within the present dataset, this population-wide morphology change is therefore the better-supported primary structural contribution to the SP5 → FP5 improvement, although local nanoscale heterogeneity and possible surface modifications may also contribute.

The flake geometry improves absorption through two complementary effects. Its high aspect ratio modifies the demagnetizing field and magnetic anisotropy while increasing the effective area available for polarization. At the same time, the derived electromagnetic parameters show that FP5 combines a markedly larger attenuation constant with near-unity normalized input impedance at 16.9 GHz. The strong RL minimum therefore reflects a favorable balance between wave entry and internal dissipation: strong loss is useful only when reflection at the absorber surface is sufficiently suppressed.

Ball milling also introduces local nanoscale structural changes. HRTEM/SAED reveals approximately 5 nm band-like regions containing limited nanocrystalline order within the amorphous matrix, while the Cole–Cole response of FP5 shows a broader distribution of dielectric relaxation. These local features may provide additional polarization or magnetic-pinning sites, but their bulk contribution cannot be quantified from the present microscopy. Surface defects or a thin oxide layer formed during milling may also contribute to the dielectric response and cannot be excluded without surface-sensitive characterization.

The frequency dependence of C_0_ further indicates that magnetic attenuation cannot be described by eddy-current loss alone. Resonance-related processes are also likely to contribute, although separating natural and exchange resonance would require dedicated ferromagnetic-resonance measurements. The present study uses one controlled milling condition to establish the SP5 → FP5 structural transition; a systematic milling-parameter study would be required to map the full process window. Likewise, independent conductivity, XPS, and additional region-resolved TEM measurements would help quantify the individual contributions suggested by the present results.

## 5. Conclusions

- Pulse-discharge atomization produced predominantly amorphous Fe_85_P_9_C_2_B_2_Si^2^ spherical powders over a broad size range, enabling particle-size effects to be examined within a single alloy composition.
- Controlled wet-ball milling transformed SP5 into FP5 microflakes with thicknesses of approximately 0.4–1.4 μm and lateral dimensions of 8–18 μm while largely retaining the amorphous matrix. Local approximately 5 nm band-like heterogeneous regions containing limited nanocrystalline order were also observed.
- FP5 exhibits enhanced dielectric and magnetic attenuation. At 16.9 GHz and 1.7 mm, α reaches 235.9 m−1 and |Z_in_/Z_0_| is 0.999, showing that the deepest absorption minimum results from the combination of strong intrinsic attenuation and favorable impedance matching. Cole–Cole and C_0_ analyses indicate distributed dielectric relaxation and show that eddy-current loss alone cannot explain the magnetic attenuation.
- FP5 achieves a minimum RL of −53.5 dB at 16.9 GHz with a matching thickness of 1.7 mm and an EAB of 6.2 GHz at 1.85 mm. Within the present dataset, the high-aspect-ratio morphology is the better-supported primary structural contribution to the improvement, while local nanoscale heterogeneity may provide an additional contribution.

## Figures and Tables

**Table 1:** Comparison of the microwave absorption properties of representative Fe-based absorbers.

Microwave Absorber	Structure/Design Strategy	Filler Loading (Matrix; as Reported)	Matching Thickness (mm)	Matching Frequency (GHz)	Minimum RL (dB)	EAB (GHz, RL ≤ −10 dB)	Ref.
FeSiBCuNb amorphous alloy	Fe-based amorphous alloy/composition optimization	40 (paraffin)	2.0	11.6	−27.3	4.2	[26]
Fe_80_B_20_ amorphous alloy	Fe-based amorphous alloy/magnetic loss regulation	50 (epoxy resin)	2.0	4.2	−39.4	5.0	[5]
FePCBCu amorphous-nanocrystalline alloy	Amorphous/nanocrystalline-graphite sheet structure	60 (paraffin)	1.6	15.84	−67.95	5.36	[30]
FeCrMoNiPBCSi amorphous alloy	Fe-based amorphous alloy/alloy design	30 vol.% (silicone)	3.55	7.08	−60.3	2.3	[2]
CIP@SiO_2_@Mn_0.6_Zn_0.4_Fe_2_O_4_	Core–shell–shell interface engineering	As reported	2.0	11.57	−44.24	7.12	[31]
Amorphous Co_100−x_Fe_x_ nanoparticles	Amorphous magnetic nanoparticles/nanoscale morphology control	50 (paraffin)	2.6	13.6	−42.0	5.6	[16]
FeSiCr@ferrite heterointerface composites	Interface engineering	50 (paraffin)	2.1	12.8	−64.13	8.72	[25]
FePCBSi amorphous alloys (FP5)	Amorphous microflake + deformation-induced heterogeneous regions	50 (paraffin)	1.7	16.9	−53.5	6.2 (1.85 mm)	This work

Several reported Fe-based absorbers achieve lower minimum RL values or broader bandwidths through composition modification, carbon incorporation, or multilayer heterointerface design. The present FePCBSi microflakes instead obtain a competitive combination of strong attenuation, a 1.7 mm matching thickness, and broad Ku-band coverage within a single Fe-based amorphous alloy composition. This comparison highlights morphology control as an effective complement to composition and interface engineering.

## Data Availability

The original contributions presented in this study are included in the article/Supplementary Material. Further inquiries can be directed to the corresponding authors.

## Supplementary Materials

The following supporting information can be downloaded at https://www.mdpi.com/article/10.3390/nano16191242/s1. Figure S1. Additional electromagnetic-loss analyses: (a) Cole–Cole plots derived from the measured complex permittivity; (b) frequency dependence of the eddy-current coefficient C_0_ = μ″/(μ′^2^f).
