# Supplementary material for: Fe-Based Amorphous Microflakes with Deformation-Induced Nanoscale Heterogeneity Prepared by Pulse-Discharge Atomization and Ball Milling for Broadband Microwave Absorption
Source: Nanomaterials (Basel). 2026 Oct 1;16(19):1242. doi: 10.3390/nano16191242 (PMC13666872; doi:10.3390/nano16191242)
Supplement: Supplementary file 1 [file nanomaterials-16-01242-s001.zip › nanomaterials-4579153-supplementary.pdf]

Supplementary Materials

Fe-Based Amorphous Microflakes with Deformation-Induced Nanoscale Heterogeneity Prepared by Pulse Discharge Atomization and Ball Milling for Broadband Microwave Absorption

Supplementary Figure S1

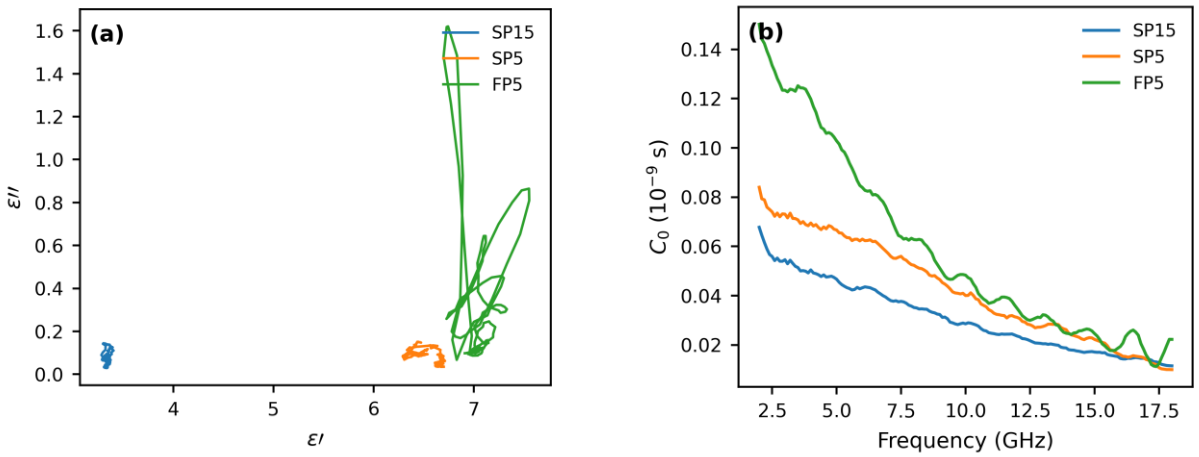

Figure S1. Additional electromagnetic-loss analyses: (a) Cole–Cole plots derived from the measured complex permittivity; (b) frequency dependence of the eddy-current coefficient $C_0 = \mu''/(\mu'^2 f)$.

The distorted and multiple high-frequency Cole–Cole trajectories of FP5 indicate a broader distribution of dielectric relaxation processes after flake formation. These trajectories support the presence of distributed relaxation but do not uniquely identify their microscopic origin. The pronounced frequency dependence of C_0 for all three samples shows that eddy-current loss alone cannot account for the magnetic attenuation over the measured band; resonance-related processes are therefore also likely to contribute. The Cole–Cole analysis is derived directly from the measured ε′ and ε″ data. Because the effective electrical conductivity of the powder/paraffin specimens was not independently measured, Figure S1a is not used to quantify conduction loss.
